# Promoting the Circular Economy on an Island: Anaerobic Co-Digestion of Local Organic Substrates as a Possible Renewable Energy Source

**DOI:** 10.3390/microorganisms11020285

**Published:** 2023-01-21

**Authors:** Juana Fernández-Rodríguez, Mónica Di Berardino, Santino Di Berardino

**Affiliations:** 1 Department of Chemistry, Instituto de Biodiversidad y Medio Ambiente (BIOMA), University of Navarra, 31080 Pamplona, Spain; 2 Higher Technical Institute (IST), 1000-001 Lisbon, Portugal; 3 Bioenergy Department, Energy and Geology National Laboratory (LNEG), 1000-001 Lisbon, Portugal

**Keywords:** anaerobic co-digestion, biogas, mesophilic BMP, organic waste, island, kinetic, local circular economy

## Abstract

The local waste co-digestion is an interesting option to tackle in reduced and isolated areas like the islands. The islands have limited territory and scarce fuel production. Moreover, organic waste can create serious environmental problems in soil, water and air. Anaerobic co-digestion (AcoD) is a technology fulfilling the concept of waste-to-energy (WtE) based on local resources. The valorisation of organic waste through AcoD on an island would prevent environmental impacts, while being a source of renewable energy. In this study, cow manure (outdoor and indoor), pig slurry, bird manure, kitchen waste, sewage sludge and oily lacteous waste produced on Island Terceira (Portugal) were tested in mesophilic −35 °C- Biochemical Methane Potential (BMP) co-digestion assays. The goals were to analyse the recalcitrant and high potential produced waste and to estimate the energetic supply source on the island. The cow manure and pig slurry were used as inocula and specific methanogenic activities (SMAs) were carried out. The results showed that both substrates have a significant methanogenic activity–SMA 0.11 g-COD/(g-VSS.d) and 0.085 g-COD/(g-VSS.d), respectively. All the studied combinations were feasible in AcoD, showing TS removals in the range of 19–37%; COD removals in the range 67–78% and specific methane yields from 0.14 to 0.22 L/gCOD removed, but some differences were found. The modified Gompertz model fitted the AcoD assays (R^2^ 0.982–0.998). The maximum biogas production rate, $R_{max.}$ was highest in the AcoD of Cow+Pig+Oily and in the Cow+Pig+Sludge with 0.017 and 0.014 L/g-VS_added_.day, respectively, and the lowest in Cow+Pig+Bird with 0.010 L/g-VS_added_. In our AcoD studies, the bird manure limited the performance of the process, since it was recalcitrant to anaerobic degradation. On the other hand, the oily lacteous waste showed a great potential in the anaerobic digestion. The estimated biogas production, from the best-studied condition, could cover the 11.4% of the energy supply of the inhabitants. These preliminary results would prevent the environmental impact of organic waste on the island and promote the use of local waste in a circular economy scenario.

## 1. Introduction

Islands are limited and isolated spaces in which technological solutions have to be investigated to prevent environmental damage. Different technologies for water [1], or energy supply have been proposed [2] due to island energy systems showing weak connection to the mainland, and congestion and stability problems. In terms of waste, the accumulation on landfills can create environmental problems in isolated spaces. In fact, the loss of biodiversity or the land occupation, between others environmental impacts, can be more remarkable in small spaces. In addition, burial waste implies a loss of nutrients that could be valuable in recycling processes.

On the other hand, carbon-neutral scenario is the main challenge for the environmental current legislation in European Commission. Indeed, the Decision 2011/753/EU [3] declared the anaerobic digestion (AD) of organic wastes as a green technology since it promotes an energetic and agronomic valorisation of residues. Recently, the European Commission has published a communication focusing on energy recovery from waste, emphasising the favourable role that AD can play in the circular economy [4] as producer of renewable energy, reducer of odours and contributor to greenhouse emissions control. The objectives set out by the Energy Union Strategy and the Paris Agreement were focused on waste-to-energy (WtE), reinforcing the use of AD in waste treatment to produce a gaseous biofuel to reduce the carbon emissions.

AD is microbiological process in which a consortium of bacteria and archaea transforms the organic substrates in the absence of oxygen. AD is based on four microbiological stages: hydrolysis, acidogenesis, acetogenesis and methanogenesis. In each stage, different groups of microorganism can be involved [5]. Accordingly, AD changes the organic matter from the substrate into a methane-rich biogas, a renewable fuel useful for heating, electric energetic generation or for replacing natural gas, after purification and concentration to biomethane [6]. Biogas production from organic waste is considered a promising alternative to fossil fuel based energy. AD is an eco-friendly process considered a key-factor for sustainability. This process contributes to sustainable management of organic waste since the process reduces greenhouse gas emissions [7]. In addition, the digestate is biologically stabilised, and hence suitable for composting processes. Furthermore, the anaerobic degradation of the feedstock releases the inorganic nutrients (ammonia, phosphate, potassium, etc.) in ionic form, which is chemically suitable for land fertilisation, even in hazardous sewage sludge [8]. In the overall process, the solubilisation of solids organic substrates as the first stage is a key factor in tackling problems related to AD, which is especially relevant in complex materials [9]. In this context, different temperatures can be applied to the process based on the anaerobic microorganisms that grow in a wide range of temperatures, from −5 °C to 80 °C, and show significant differences in the speeds of the biochemical reactions [10,11]. In general, mesophilic (25–45 °C) is the widespread temperature range in anaerobic digestion at industrial scale because its more cost-effective: higher stability and lower energy expenditure.

Anaerobic co-Digestion (AcoD) of several organic substrates is a beneficial solution since the nutritional shortcomings of isolated substrates are covered. The AcoD can balance the overall process because of the different velocities of the microbiological stages [12]. Consequently, AcoD provides a more stable operation than monodigestion. At higher scales, AcoD improves the anaerobic digestion process and makes its setup more feasible [13]. Additionally, some substrates can be recalcitrant to anaerobic degradation in monodigestion. In this way, bird manure has been quite unpopular due to the high nitrogen and lignocellulose content [14]; AcoD could be a promising configuration to valorise it. Furthermore, new configurations of the AcoD have been studied; for example, sometimes at lab scale, cattle waste was used only as a source of inoculum to start up the anaerobic digestion, but currently authors are investigating the benefits of AcoD as substrate at once [15]. The use of organic local substrates as substrate and inoculum, at the same time, tackle the anaerobic digestion in isolated places, and don’t depend on external effluents as source of inoculum.

Based on the benefits of AcoD, several studies have been published dealing with organic waste from agroindustrial activities in codigestion. In this sense, the AcoD of MSW (Municipal Solid Waste) with purifying mud has been studied [16,17]. The AcoD with other waste showed successful results in the use of municipal solid waste, which included sludge, cow manure or waste from other animals [18]. The waste can come from different sectors, like the alcoholic beverage industry [19], which showed a worthy performance in the overall process. The AcoD of waste has advantages such as higher specific rate of methane production, higher removal rates of organic matter in the waste or better dewaterability of the final effluent in the process, among others benefits. A recent review [20] has analysed that AcoD is a favourable management of kitchen waste and animal manure, since the biogas yield improved. Additionally, the AcoD of food waste with animal manure not only provides good buffering capacity to the AD systems, but the nutrient profile is also favourably transformed [21,22].

Nevertheless, the presence of inhibitors, the low biodegradability of some substrates and the instability of the process (ammonia or VFA accumulation) are challenges to deal with. The operational parameters like mixing ratio, effect of pH, C/N ratio or VFA concentration should be adjusted to get the maximum biogas yield. Furthermore, the addition of waste and sludge in the digester presents operational and economic complications due to substrate dilution, the organic load and the transport of waste, which cannot justify the additional investment required. Hence, the need for characterisation studies of AD at small scales results is essential for proper management, with special relevance in the isolated and small places, like islands. Islands have limited territory and scarce fuel production and the local waste AcoD can report remarkable benefits. The optimization of the AcoD with local waste is a challenge; to minimise the environmental impact of the waste, and at the same time, the recovery of energy (from the biogas rich in methane) could cover one part of the local demand. Local solutions should be investigated to solve local problems.

In this regard, this study is focused on the evaluation of energy supply from AcoD of the main organic substrates generated on Terceira Island, Portugal. This paper mainly evaluates the residual biomass produced on the island, the biodegradability of the organic waste generated, the potential for bioenergy (the maximum biogas yield) and the removal of organic matter in the digestate, based on the co-digestion of local organic waste from the main industries on the island. The activity tests were carried out to assess the inocula activity and mesophilic Biochemical Methane Potential (BMP) was carried in AcoD. Finally, the potential for waste-to-energy (WtE) from the point of view the methane produced was estimated, as a proposal for energy recovery.

## 2. Material and Methods

### 2.1. Local Organic Wastes

The organic wastes used in the study come from the main industry activities on Terceira Island, Portugal. Terceira Island in Azores, Portugal is the second most inhabited island of the Azores, with 54,874 residents [23]; it has 401.9 km^2^ of surface, 30.1 km long and 17.6 km at its widest point. The economy of the island is based mainly on agriculture and livestock sectors. Indeed, the industries associated with the processing of dairy products have high relevance in the local economy. The cattle raising consists mainly of animals of the Holstein–Friesian breed called the Dutch cattle, although it is worthy to note the native breed Big Branch.

The organic substrates came from the main economic activity on the island. Specifically, the substrates used in this research were:

- Waste from dairies collected in a dairy farm. Two different dairy wastes were collected: from outdoors places–cow manure 1 (C1)- and from coverage places–cow manure 2 (C2).
- Solid waste from a kitchen of canteens military–air base of Lajes (K).
- Waste from pig farms–pig slurry (P).
- Waste from aviaries industries –bird manure (B). Poultry manure (chickens, geese, turkeys and ducks).
- Sewage sludge from a local wastewater treatment plant –WWTP- (S).
- Waste from a treatment plant of lacteous products, called Pronicol. It was oily lacteous waste from the flotation tank- (O).

The different organic waste were collected and frozen at −20 °C. They were transported and storage in the Energy and Geology National Laboratory (LNEG) in Lisbon, Portugal. In the labs of LNEG, the characterisation and the experimental setup were carried out.

The inocula used were the pig slurry (P) and the cow manure (C2) since previous studies have shown they contain all the microbiological population to carry out AD [24,25]. These wastes contain, apart from the organic content, an important methanogenic population present in the intestinal tract; therefore, these substrates have been used as inoculum for biochemical methane potential (BMP) assays. An acclimatisation stage was previously completed: defrozen at room temperature and, lately, 72 h at 35 °C, the working temperature. Additionally, in this work activity tests were performed before the AcoD experiments, in order to verify the methanogenic activity of both inocula.

### 2.2. Analytical Determinations

Samples were analysed according to the *Standard Methods for the Examination of Water and Wastewater* [26]. The analytical parameters studied have been: total solids (TS), volatile solids (VS), total suspended solid (TSS) and volatile suspended solids (VSS) using thermogravimetric methods. Furthermore, chemical oxygen demand (COD) (by dichromate potassium digestion and titration), soluble total organic carbon (TOC_s_ with a TOC-VWS Shimadzu analyser equipped with non-dispersive infrared –NDIR- sensors); Total Kjeldahl nitrogen (TKN, by digestion, distillation and titration), proteins (based on the content of organic nitrogen), phosphorus (by digestion and spectrophotometry), pH and fat (extraction at 60 °C) were calculated.

The concentrations of volatile fatty acids (VFAs) (acetic, propionic, butyric, isobutyric, valeric and isovaleric), at the beginning and at the end of the tests, were determined by gas chromatography using a Hewlett Packard gas chromatograph (model 5890) equipped with a flame ionization detector (FID). The biogas samples were analysed in a gas chromatograph (Varian 3380), equipped with a detector thermal conductivity detector (TCD) and two columns: Porapack S and a molecular sieve, for the quantitative determination of the biogas composition. The method was developed with an external standard. The carrier gas was helium.

### 2.3. Experimental Setup

#### 2.3.1. Activity Tests: Specific Methanogenic Activity (SMA)

**Figure 1:**
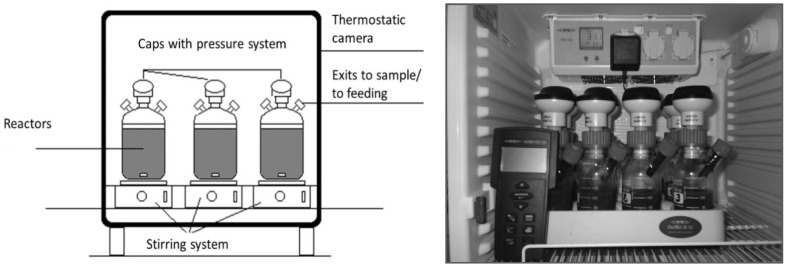
Scheme and picture of the system used in the activity tests.

Laboratory tests were carried out in batch reactors to assess the specific methanogenic activity (SMA) of the inocula: cow manure and pig slurry. Equipment based on a pressure transducing system, with caps of pressure sensors -WTW OxiTop^®^-C IS-, was used (Figure 1). An infrared reader was employed to get the information from the pressure sensors.

Different concentrations of sodium acetate: 0.5 g/L, 0.75 g/L and 1.5 g/L were used [27,28]. Fundamentally, acetoclastic bacteria are responsible for 70% of the methane produced in the anaerobic digestion process; consequently, the study of acetoclastic activity can estimate the inoculum activity. The reactors were purged with nitrogen (inert atmosphere) and sealed. For each concentration of sodium acetate, three tests were carried out. Additionally, two tests without substrate (the blanks of the experiment) were studied.

The results were analysed based on the simplified equation of zero order kinetics, without growth, Monod model results. The expression is shown in Equation (1):

(Ac)CH_4_ = (dV CH_4_/dt)/(X_0_.V_r_.f) (1)

where

(Ac) CH_4_ is the methanogenic activity (gCOD(mLCH_4_).g-1VSS.d-1);
V CH_4_ is the amount of methane produced (mL);
t is the reaction time (days);
X_0_ is the inoculum concentration (g/mL);
V_r_ is the effective volume in the reactors (mL);
f is the conversion factor that represents the amount of COD in grams per mL.

In the linear region of the trend, the slope can estimate the speed of the process. The linear regression was adjusted using Excel.

#### 2.3.2. Biochemical Methane Production (BMP) Tests

The BMP tests were settled following up the recommendations of Holliger et al. [29] for the inoculum and substrate concentrations. The assays were carried out to avoid the accumulation of acids in the reactor: the inoculum was feeding according to 5 g-VSS/L (VSS: Volatile suspended solids) and the organic loading was 5 gCOD/L (COD: Chemical oxygen demand), to prevent excessive production of volatile acids and the inhibition of the system. The BMP was carried out in wet anaerobic conditions (4–10% TS-total solids).

**Figure 2:**
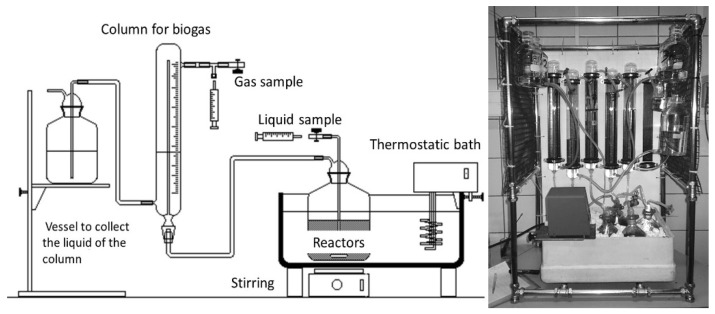
Scheme and picture of the used equipment in BMP tests.

Batch anaerobic reactors were used to study the BMP in AcoD. The system consisted of 14 reactors, with 1 L total volume and 0.8 L working volume. The scheme of the configuration is shown in Figure 2. The reactors are set in a thermostatic bath to maintain a constant temperature. The assays were carried out at a temperature of 35 °C (mesophilic range). The production of biogas was collected and measured by water displacement. Graduated tubes were placed on top of reactors, which were connected to a reservoir measuring the produced biogas. The tubes were filled with a saline and acid solution composed of H_2_O (79%), NaCl (20%) and HCl (1%), to minimize the CO_2_ dissolution in the liquid. During the tests, the produced biogas generated a pressure that displaced the fluid in the graduated columns towards the reservoir, in a proportional amount to the generated volume. The biogas was measuring periodically by the displaced volume in standard conditions for temperature and pressure. The samples of biogas were collected using gas syringes.

The Table 1 shows the AcoD ratio in the experimental design. All the assays included the combination of both substrates/inocula (cow manure+pig slurry) and the addition of one organic substrate: bird manure, kitchen waste, sewage sludge or oily lacteous waste, to estimate the individual effects on the overall AcoD. The assays were performed in duplicate. Wastes were diluted in a proportion of 1:1- cow manure from outdoors places, pig slurry and bird manure- and 1:3- cow manure from covered places due to the concentration of organic matter. Additionally, tap water was added to the working volume (0.8 L). The final dilution factor in the reactors was approximately 1:2. The pH was adjusted to 7.0 and the reactors were purged with a nitrogen stream for 5 min to remove all the oxygen in the headspace, and set the anaerobic conditions inside the reactors (inert atmosphere). The systems were closed and placed in a thermostatic bath, being shaken by hand daily.

The cow manure 2 (C2), due to the major concentration of methanogenic population (see the Section 3.2. Specific methanogenic activity -SMA-), was mixed with pig slurry (P) and both were used as inoculum in a fixed proportion of 330 mL and 270 mL, respectively, to avoid a fast acidification.

### 2.4. Kinetic Analysis: Modified Gompertz Model

Commonly, the modified Gompertz model can be applied to find the relationship between cumulative biogas production (y(t):L/g VS) and the minimum time to produce biogas (t: days), using kinetic parameters. The expression is shown in Equation (2):

(2) $$y\left(t\right)=y_{m}.\exp\left\{-\exp[\frac{R_{max.}}{y_{m}}e.\left(\lambda -t\right)+1]\right\}$$

where

y(t) is the cumulative biogas yield (L/g) at a time t (days).

$R_{max.}$ is the maximum specific biogas production rate (L/(g-VS.day))

$y_{m}$ is the maximum biogas yield potential (L/g-VS)

λ is the lag-phase time (days)

e is a mathematical constant (2.71828)

The model was fitted to those experimental results related to the production of biogas, since previous studies have shown this to be an effective way to describe the kinetic of anaerobic codigestion [30,31,32,33].

## 3. Results and Discussion

The results and their discussion are presented under four sections, for the sake of clarity: characterisation of local waste, specific methanogenic activity of the inocula, BMP in AcoD tests and evaluation of energy supply, based on the methane potential on the island.

### 3.1. Waste Characterisation and Analysis

The Table 2 shows the characterisation of the wastes used in this study. The analytical parameters were carried out in duplicates. The bird manure was the substrate with highest TS content with 66%TS compared to the pig slurry with barely 7%. The bird manure is too concentrated in TS; consequently, according to the bibliography [25], the waste should be managed in dry anaerobic processes. In the case of kitchen waste, the concentration of TS is more than 27%, a value slightly lower for this type of waste [20], which is related to the raw material used in the production of food. The VS was high in all the substrates, between 80 and 95% over the TS concentration, what represents the high organic content and suitable substrates for AD. The values of COD of the waste are in accordance with the TS and VS’ values. The maximum value of COD was quantified in the oily lacteous waste (335 g/L) versus the minimum concentration in pig slurry (47 g/L). The highest COD found in the oily waste can suggest the potential of this waste in the experimental setup. In fact, the lipids are an important source of methane since they can produce until 3 times more biogas than protein or carbohydrates [34]. The organic nitrogen is related to the proteins. The different concentration in total organic nitrogen (between 80 and 700 mg/L) and carbon (between 4 and 27 gTOC_s_/L) of different substrates set the AcoD as a balanced nutritional supply for the anaerobic microorganism.

The characterisation showed that the waste have a suitable composition for the application in AcoD. However, following the references to start-up the BMP, the high concentration of organic matter has implied an extra dilution of some of them. The cow manure, the pig slurry and the bird manure were diluted in a proportion of 1:1 and the oily lacteous waste was diluted 1:3, as explained in Section 2.3.2.

### 3.2. Specific Methanogenic Activity (SMA)

**Figure 3:**
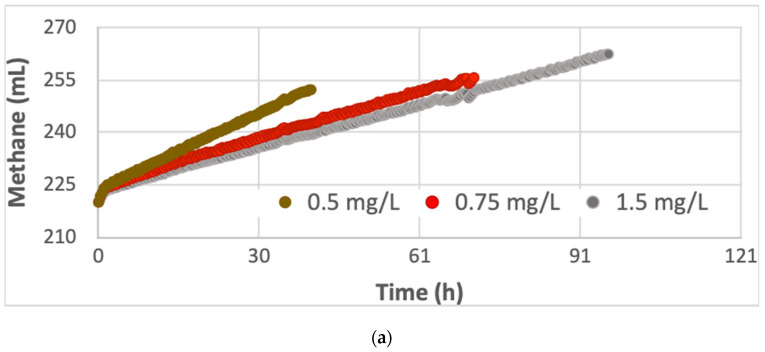

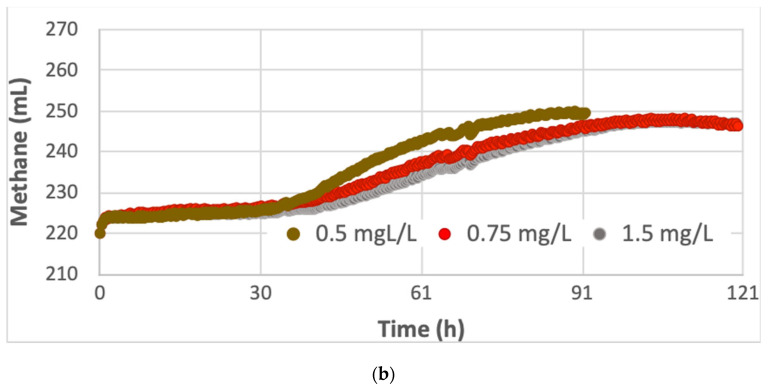
Cumulative methane production (mL) in the activity tests for the inocula: (**a**) cow manure, (**b**) pig slurry, at different concentrations of sodium acetate: 0.5, 0.75 and 1.5 mg/L.

The specific methanogenic activity (SMA) of pig slurry and cow manure was tested at different concentrations of sodium acetate: 0.5 g/L, 0.75 g/L, 1.5 g/L [27,28]. Furthermore, two blanks were carried out. Figure 3 shows a representation of the cumulative methane production (a- cow manure; b- pig slurry), once the average of the blanks was subtracted.

Both inocula showed a high acetoclastic SMA in less than 83 h (even 33 h in the case of cow manure). The final acetic acid concentration was zero in all assays. The methanogenic activity for different concentrations of sodium acetate was determined according to the initial substrate concentration and the methane produced during the reaction according to the Monod model Equation (1).

The cow manure showed a higher SMA than pig slurry, SMA = 0.11 versus SMA = 0.085 gCOD/(g-VSS.d) (Table 3), respectively, probably due to the biodiversity and composition of methanogenic populations in the rumen of cows. Both results are in agreement with the literature, which referred the methanogenic activity in ranges from 0.020 to 0.200 gCOD/(g VSS.d^−1^) [27]. Consequently, the results indicate that both inocula showed a methanogenic activity in active conditions for the biodegradability tests.

Additionally, a regression was adjusted in the linear region. The slope can give an idea of the process speed. The highest velocity was reported at the lowest concentration of sodium acetate (0.5 g/L) in both inocula.

The final composition of the biogas, 75% methane, in activity tests showed that pig slurry and cow manure had a methanogenic activity in the studied period. However, the velocity for cow manure was higher comparing to pig slurry, with a lag phase of 40 days (Figure 3a,b). According to these results, both substrates could be suitable as inocula, but the cow manure could improve the AD performance.

### 3.3. BMP Tests

Five AcoD combinations and two inocula were assessed to investigate the mesophilic BMP performance of the local waste. The inoculum-to-substrate ratio I:S did not show biomass inhibition [35], perhaps due to the high organic content of the inoculum. The reactors were running for 60 days to assure the total degradation of the waste under the selected experimental conditions. Different parameters were analysed to characterise the performance of AcoD. This section is divided in three subsections: effects on the digestate, production of biogas and kinetic analysis.

#### 3.3.1. Effects on the Digestate: Removal of Organic Material from AcoD of Local Waste

The organic content of digesters decreased considerably in 60-days operation, as expected. Different parameters in the digestate were analysed: TOCs, Kjeldhal nitrogen, proteins, phosphorous, COD, TS and VS (Table 4).

In general, the total organic matter removals in AcoD were noteworthy: between 20 and 37% -VS and between 66 and 77% for COD. In respect to TS concentration, the reactors with the highest removals were those operating in AcoD pig slurry and cow manure (C+P); the reactors in co-digestion cow manure, pig slurry and oily waste (C+P+O) were around 37%. The lowest result was the obtained in the co-digestion of cow manure, pig slurry and sewage sludge (C+P+S), which was barely 20%. However, the maximum removal rate of COD was obtained in this last condition (C+P+S) with more than 77%, indicates that the majority proportion of the organic matter in the reactor was in the soluble form, which is more accessible for the microorganisms. The other conditions reached values between 67 and 70% of COD removal. Similar results, between 70.3–85.9% of COD_rem_, were reported in the AcoD of poultry litter and wheat straw [36]. Moreover, Xing et al., [37] found COD removal efficiencies of 64–79% in a mesophilic AcoD of waste activated sludge and food waste. This good performance is likely due to the hydrolysis and biodegradation of an important portion of the suspended solids.

The final COD indicates that there is a remaining quantity of organic substrate to be degraded. Therefore, the difference between the total COD and TOC_s_ shows that most of the soluble organic matter was degraded, and the remaining organic fraction is included in the solid digestate. However, due to the long length of the experiment (60 days), it could be concluded that the remaining suspended organic material presents low biodegradability in the current operational conditions.

The reduction of organic nitrogen is remarkable, between 55–70%, which is likely indicative of the degradation proteins in the anaerobic process. Additionally, the decrease in organic nitrogen should increase the ammonia nitrogen [38].

In general, all the AcoD assays have shown a generation and subsequent degradation of the Volatile Fatty Acids (VFAs), revealing the good state of acetoclastic bacteria and the methanogenic Archaea consortium [39]. The values of pH at the end of the process, around 7.5 in all the AcoD, indicated that the process was in the best condition, having high stability and ability to support overloads [40].

#### 3.3.2. Generation of Biogas from AcoD of Local Wastes

**Figure 4:**
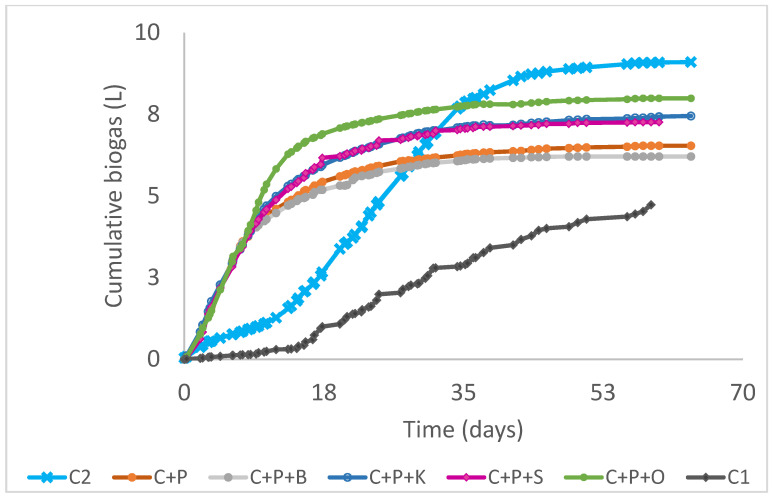
Evolution of the cumulative biogas production (L). Being C2: Cow manure 2; C+P: Cow manure+Pig slurry; C+P+B: Cow manure+Pig slurry+Bird manure; C+P+K: Cow manure +Pig slurry+Kitchen waste; C+P+S: Cow manure+Pig slurry+Sewage sludge; C+P+O: Cow manure+Pig slurry+Oily lacteous waste; C1: Cow manure 1.

The biogas composition was periodically characterised by gas chromatography. The analysis of cumulative biogas, in terms of time and per unit of organic matter, is shown in Figure 4 and Table 5. In all the assays, the performance of the duplicates showed less than 5% the coefficient of variation -CV- and the average of the different conditions was carried out. For the sake of clarity, one trend for each condition was represented.

The biogas production was different according to the substrate, but all the cases showed a similar trend. At the beginning, the initial stage corresponds to a logarithmic growth of acidogenic bacteria. This increase in cumulative biogas production can be related to the protein degradation [41].

The results indicate the adaptation of inoculum to the organic substrates. The lag phase lasted less than 10 days in C1 and C2, and no lag phase in the rest of conditions was detected. The production of biogas from the first day of operation showed the correct acclimation of the inoculum to the substrates. Renggaman et al., [33] reported that the AcoD can reduce the lag phase, between 3–8 days, comparing to the anaerobic degradation of isolated waste: slaughter waste and animal manure.

**Figure 5:**
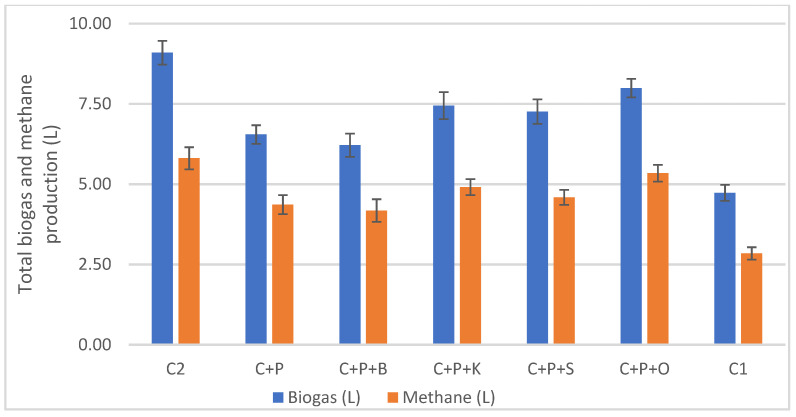
Biogas and methane volumes (in L) from the co-digestion tests. Being C2: Cow manure 2; C+P: Cow manure+Pig slurry; C+P+B: Cow manure+Pig slurry+Bird manure; C+P+K: Cow manure+Pig slurry+Kitchen waste; C+P+S: Cow manure+Pig slurry+Sewage sludge; C+P+O: Cow manure+Pig slurry+Oily lacteous waste; C1: Cow manure 1.

The composition of biogas, referred to methane, is shown in the Figure 5. The nitrogen was removed from the composition, because it was added at the beginning to produce the inert atmosphere. Finally, the biogas composition was 70% CH_4_ and 30% CO_2_, approx. (Table 5). The analysis of the biogas composition in respect to the time showed that the methane appears from the beginning, with a concentration around of 60%, which confirms the methanogenic activity of the inoculum (Section 3.2). Subsequently, the methane concentration was increased with values close to 78% at the end. The higher accumulation of the carbon dioxide in the liquid-phase, being much more soluble compound than methane, and the pH that changed the carbon balance inside the reactor (buffer balance) can be the reasons of those values. The release of ammoniac nitrogen from the degradation of the proteins [38] induced a slight decrease of the pH that tends to be compensated by the dissolution of carbon dioxide in liquid phase, which forms carbonic acid. The methane production decreased in the final stage, probably due to the diminution of the biodegradable substrate, which increases the consumption for cellular maintenance and causes a slight increase in the CO_2_/CH_4_ ratio.

The highest accumulated biogas in AcoD assays was recorded in the cow manure, pig slurry and oily waste (C+P+O), with 8 L in 60 days, corroborating the potential of the lipids to produce biogas comparing to other substrates like proteins or carbohydrates [34]

The lowest value in AcoD was reported in the combinations of cow manure and pig slurry (C+P) and in the cow manure, pig and bird manure (C+P+B), obtaining barely 6 L-biogas. Our results confirm that the bird manure has shown to be refractory to anaerobic biodegradation.

#### 3.3.3. Kinetic and Yield Analysis from AcoD of Local Waste

The kinetics of the biogas production and the yield of methane production in the AcoD assays, considering the COD consumed in the process, are shown in Table 5. Assuming that the maximum theoretical conversion factor of methane production per COD removed is 0.350 LCH_4_/gCOD_rem;_, the highest specific methane yields was reported in the AcoD of cow manure, pig slurry and kitchen waste (C+P+K), 0.224 L/g COD_rem_ and the cow manure from coverage places (C1) with 0.236 L/g COD_rem_. Xing et al., [37] observed a methane yield of 0.26–0.33 mL/gCOD in a mesophilic AcoD of waste activated sludge and food waste.

However, the minimum specific yields of methane were achieved in the AcoD of cow manure with pig slurry and sewage sludge (C+P+S) with 0.141 L/g COD_rem_ and cow manure from outdoors places (C1) with 0.084 L/g COD_rem_. With respect to the specific methane yield expressed as L/g-VS_added_, the highest yield was reported in the C+P+O codigestion with 0.162, compared to the lowest yield in the C+P+B 0.126 L-methane/g-VS_added._. Similar results (0.10–0.19 L/g-VS_added_) were obtained by Zhan et al. [36] in a AcoD of poultry litter and wheat straw study.

The kinetic analysis demonstrated that the modified Gompertz model was suitable to describe the AcoD process. The model fitted all the experimental conditions, with the coefficient of determination R^2^ ranging from 0.9822 to 0.9979. The kinetic parameters from the total cumulative biogas obtained in this study are in agreement with the reported values in previous papers [42]. The specific biogas yield potential, $y_{m}$, was the highest in the C+P+S and in the C+P+O (0.236 and 0.233 L/g-VS_added_, respectively) compared to the other AcoD combinations. The lowest results were reported in C+P+B with 0.151 L/g-VS_added_, showing that the bird manure is a refracting waste to treat through anaerobic digestion due to the high nitrogen and lignocellulose content [14]. Similar trends were observed in the maximum biogas production rate, $R_{max.}$. The highest values were found in the C+P+S and in the C+P+O (0.014 and 0.017 L/g-VS_added_.day, respectively) and the lowest in C+P+B with 0.010 L/g-VS_added_. With respect to to the minimum time to produce biogas, the lag time, λ, was approximately 0 days in all conditions. Suitable acclimation of the inoculum and the appropriate methanogenic activity (as seen in Section 3.2. Specific methanogenic activity) promoted fast production of biogas and no time was needed to start biogas production. The AcoD with oily lacteous waste showed a slightly longer time, 0.9 ± 0.14 days, likely due to the fatty nature of the substrate, which makes it difficult to access the microorganism for waste. The lag phase in the case of inocula isolates was in the range 8.31 and 11.84 days, revealing one of the advantages of the AcoD.

These results should be analysed depending on the composition of the waste. In literature, the methane production of the three general groups of organic compounds can be found: carbohydrates, proteins and lipids, which are 0.4, 0.5 and 1.0 m^3^ CH_4_/kg VS, respectively [43]. Thus, it can be observed that the good performance in the AcoD with cow manure, pig slurry and oily lacteous waste (C+P+O) could be due to the high potential of the fats to generate methane.

### 3.4. Evaluation of Methane Potential on the Island

Biogas has been recognised as a source of renewable energy available to promote the socio-economic development [44]. It can be valorised as a replacement of natural gas after upgrading processes. In developing countries, biogas is commonly used in household activities like cooking and lighting. Nevertheless, the application in developed countries is focused on commercial industrial uses [45]. In Table 6, the simulation of waste-to-energy (WtE) based on the methane production according to the studied waste can be evaluated. The calorific value of biogas was estimated according to an enrichment of 70% methane (as calculated in the Section 3.3.2) as 5500 kcal/m^3^. Based on the main results in this study, the production of biogas could cover the 11.4% of energy demand for the inhabitants on the island (considering an average consumption of 10 kWh/(person-day). Consequently, a renewable energy source based on waste can be applied. The proposal could deal with several problems at once: the negative environmental impact of waste, which is especially important in isolated places such as islands, and energy recovery as an alternative to fossil fuel based energy.

## 4. Conclusions

The motivation of this work has been to tackle the AcoD of several waste sources from an island and to simulate the waste-to-energy in a circular economy scenario. The following main conclusions can be addressed: Cow manure and pig slurry from the island have shown to be suitable inoculum in anaerobic conditions. However, according to the activity tests, the cow manure showed higher acetoclastic activity than pig slurry, SMA = 0.11 and SMA = 0.085 gCOD/(g-VSS.d), respectively, and specially activated at low concentrations (0.5 g sodium acetate/L). AcoD of organic waste (cow manure -outdoor and indoor-, pig slurry, bird manure, kitchen waste, sewage sludge and oily lacteous waste) from an island were satisfactory in all the assays, with almost no lag phases, due to the acclimation of inocula, and yields from 0.224 L/g-COD_rem_ (C+P+K) and 0.193 L/g-COD_rem_ (C+P+O). The removal of organic matter was noteworthy, in general, with COD removals of 66–77% and VS removals of 20–37%.

According to the kinetic parameters from the modified Gompertz model (R^2^ 0.9822 to 0.9979), the specific biogas yield potential, $y_{m}$, was highest in the C+P+S and in the C+P+O (0.236 and 0.233 L/g-VS_added_, respectively) compared to the other AcoD combinations. The lowest results were reported in C+P+B with 0.151 L/g-VS_added_. Similar trends were observed in the maximum biogas production rate, $R_{max.}$. The highest values were found in the C+P+S and in the C+P+O (0.014 and 0.017 L/g-VS_added_.day, respectively) and the lowest in C+P+B with 0.010 L/g-VS_added_. Our results showed that in AcoD, the local bird manure was recalcitrant to anaerobic degradation and the local oily lacteous waste had a high biogas potential production.

The estimated biogas production from the best-studied condition could cover the 11.4% of the energy supply of the inhabitants. The laboratory tests carried out showed that the AcoD has shown to be an alternative and efficient technology, with a gaseous biofuel production from a sustainable management. Additionally, the results would provide the possibility to amplify the study with more combinations of organic solid wastes from the island and the option of studying a full-scale co-digestion plant in future research. On the other hand, different pre-treatments can be proposed to increase the anaerobic biodegradation of recalcitrant waste like bird manure, or promising substrates like oily waste.

In short, based on our findings, this technology would suppose an important contribution in small and isolated places, like islands, to prevent environmental impact and recover renewable energy from organic local wastes.

## Figures and Tables

**Table 1:** Description of experimental design: ratio of AcoD.

Reactors	Name	V_water_ (mL)	V_waste_ (mL)	V_cow manure 1_ (mL) (C1)	V_cow manure 2_ (mL) (C2) *	V_pig slurry_ (mL) (P) *	V_bird manure_ (mL) (B) *	V_kitchen waste_ (mL) (K)	V_sewage sludge_ (mL) (S)	V_oily lacteous waste_ (mL) (O)	TS (%)	VS (%)
**1/2**	**C2**	200	600	-	600	-	-		-	-	5.01	4.32
**3/4**	**C+P**	200	600	-	330	270	-		-	-	4.35	3.73
**5/6**	**C+P+B**	150	650	-	330	270	50		-	-	5.16	4.99
**7/8**	**C+P+K**	150	650	-	330	270	-	50	-	-	4.82	4.26
**9/10**	**C+P+S**	150	650	-	330	270	-		50		4.65	3.74
**11/12**	**C+P+O**	150	650	-	330	270	-		-	50	4.85	4.16
**13/14**	**C1**	200	600	600	-	-	-		-	-	4.89	4.64

Being C2: Cow manure 2; C+P: Cow manure+Pig slurry; C+P+B: Cow manure+Pig slurry+Bird manure; C+P+K: Cow manure+Pig slurry+Kitchen waste; C+P+S: Cow manure+Pig slurry+Sewage sludge; C+P+O: Cow manure +Pig slurry+Oily lacteous waste; C1: Cow manure 1. * The waste cow manure 2, pig slurry and bird manure were diluted 1: 2 and cow manure 1 diluted 1: 3.

**Table 2:** Characterisation of the organic waste used in the assays.

	Cow Manure 1 (C1) *	Cow Manure 2 (C2)	Pig Slurry (P) *	Bird Manure (B)	Sewage Sludge (S)	Oily Lacteous Waste (O)	Kitchen Waste (K)
**COD (g/L)**	162 ± 9	101 ± 6	47 ± 4	215 ± 14	138 ± 8	335 ± 19	228 ± 12
**TOC** **_s_** **(g/L)**	14.12 ± 1.9	8.18 ± 1.2	4.26 ± 0.9	12.59 ± 2.1	22.16 ± 2.9	27.51 ± 2.4	20.35 ± 1.5
**TKN (mg/L)**	90 ± 4	78 ± 5	78 ± 4	179 ± 10	213 ± 13	123 ± 9	790 ± 44
**Proteins (mg/L)**	560 ± 29	409 ± 20	490 ± 29	1120 ± 53	1330 ± 69	770 ± 36	4935 ± 249
**Phosphorous (mg/L)**	908 ± 48	606 ± 35	1282 ± 76	310 ± 17	1741 ± 89	2190 ± 115	2743 ± 133
**TS (%)**	15.2 ± 0.8	11.1 ± 0.7	7.2 ± 0.4	66.1 ± 3.6	10.0 ± 0.9	8.9 ± 0.6	27.3 ± 1.8
**VS (%)**	12.1 ± 0.6	8.8 ± 0.3	5.0 ± 0.4	51.5 ± 2.3	9.0 ± 0.8	8.1 ± 0.4	26.3 ± 1.7
**Moisture (%)**	84.8 ± 4.5	88.9 ± 4.31	92.8 ± 4.0	33.9 ± 1.8	90.0 ± 4.6	91.1 ± 4.8	72.7 ± 3.4

* Organic substrates and inoculum source.

**Table 3:** Values of specific methanogenic activity (SMA).

Substrate Concentration (g/L Acetate)	Cow Manure		Pig Slurry	
	Specific Metanogenic Activity –SMA-(g-COD_(CH4)_/(g-VSS.d))	Slope Linear Regression (mL/h)	Specific Metanogenic Activity –SMA-(g-COD_(CH4)_/(g-VSS.d))	Slope Linear Regression (mL/h)
0.5	0.109 ± 0.004	0.7294	0.080 ± 0.008	0.4314
0.75	0.112 ± 0.013	0.4513	0.089 ± 0.007	0.3310
1.5	0.111 ± 0.072	0.4056	0.087 ± 0.004	0.3626

**Table 4:** Initial characteristics of substrate and organic removal in 60-days operation.

	Cow Manure 2	Cow Manure+Pig slurry	Cow Manure+Pig slurry+Bird manure	Cow Manure+Pig Slurry+Kitchen Waste	Cow Manure+Pig Slurry+Sewage Sludge	Cow Manure+Pig Slurry+Oily Lacteous Waste	Cow Manure 1
	C2	C+P	C+P+B	C+P+K	C+P+S	C+P+O	C1
**Initial Characterisation**							
**TOC** **_s_** **(g/L)**	4.09 ± 0.56	2.95 ± 0.21	3.91 ± 0.68	2.99 ± 0.59	3.03 ± 0.50	4.89 ± 0.71	2.98 ± 0.16
**TKN (mg/L)**	34 ± 5	39 ± 4	34 ± 3	39 ± 4	39 ± 4	39 ± 4	31 ± 3
**Proteins (mg/L)**	210 ± 15	245 ± 25	210 ± 21	245 ± 20	245 ± 15	245 ± 25	193 ± 18
**Phosphorous (mg/L)**	227.3 ± 28.2	341.3 ± 16.3	360.7 ± 25.3	512.8 ± 18.9	450.1 ± 21.2	478.2 ± 38.0	337.5 ± 25.9
**COD (g/L)**	40.00 ± 6.20	34.55 ± 7.50	37.27 ± 5.90	34.55 ± 3.60	45.45 ± 4.50	48.45 ± 7.90	56.52 ± 8.90
**TS (%)**	5.01 ± 1.10	4.35 ± 0.90	5.16 ± 1.20	4.91 ± 0.95	4.65 ± 1.05	4.85 ± 0.95	4.89 ± 1.25
**VS (%)**	4.32 ± 0.85	3.73 ± 0.99	4.99 ± 1.11	4.26 ± 1.06	3.74 ± 0.60	4.16 ± 0.51	4.64 ± 0.39
**Organic Removal**							
**COD_final_ (g/L)**	12.73	11.27	12.18	11.67	10.18	15.09	16.55
**Removal COD (%)**	68.18	67.37	67.32	67.37	77.60	66.80	70.88
**TS_final_ (%)**	3.64	2.74	3.34	3.28	3.76	3.04	3.16
**Removal TS (%)**	27.35	37.01	35.27	27.62	19.14	37.32	35.38
**VS_final_ (%)**	3.14	2.35	3.23	2.89	3.03	2.61	3.00
**Removal VS (%)**	27.31	37.00	35.27	32.16	18.98	37.26	35.34

**Table 5:** Biogas productions and yields in AcoD assays in 60-days operation. Parameters from the kinetic modelling accordingly to modified Gompertz Equation (2).

	Cow Manure 2	Cow Manure+Pig Slurry	Cow Manure+Pig Slurry+Bird Manure	Cow Manure+Pig Slurry+Kitchen Waste	Cow Manure+Pig Slurry+Sewage Sludge	Cow Manure+Pig Slurry+Oily Lacteous Waste	Cow Manure 1
	**C2**	**C+P**	**C+P+B**	**C+P+K**	**C+P+S**	**C+P+O**	**C1**
**Biogas Production (L)**	9.10 ± 0.80	6.54 ± 0.61	6.21 ± 0.57	7.45 ± 0.39	7.26 ± 0.72	7.99 ± 0.63	4.73 ± 0.41
**CH** **_4_** **Production (L)**	6.44 ± 0.49	4.70 ± 0.56	4.44 ± 0.38	5.22 ± 0.60	4.99 ± 0.51	5.86 ± 0.62	3.40 ± 0.240
**CH** **_4_** **(%)**	70.8	71.9	71.5	70.1	68.7	73.3	71.9
**Specific Methane Yield (L/g COD_rem_)**	0.236	0.202	0.177	0.224	0.141	0.193	0.084
**Specific Methane Yield (L/g VS_add_)**	0.184	0.132	0.126	0.151	0.147	0.162	0.096
**Kinetic Parameters**							
	**C2**	**C+P**	**C+P+B**	**C+P+K**	**C+P+S**	**C+P+O**	**C1**
**R_max_** **((L-Biogas/g-** **VS_added_ day)**	0.009 ± 0.00019	0.014 ± 0.00046	0.010 ± 0.00036	0.012 ± 0.00032	0.014 ± 0.00038	0.017 ± 0.00034	0.003 ± 0.00004
**L (days)**	8.31 ± 0.29	−0.73 ± 0.28	−0.83 ± 0.30	−0.69 ± 0.25	−0.23 ± 0.23	0.9 ± 0.14	11.84 ± 0.247
**P (L-Biogas/g-V** **_sadded_** **)**	0.281 ± 0.0031	0.210 ± 0.0013	0.151 ± 0.0010	0.205 ± 0.0011	0.236 ± 0.0012	0.233 ± 0.00079	0.132 ± 0.0014
**R^2^**	0.9942	0.9850	0.9822	0.9899	0.9896	0.9954	0.9979

**Table 6:** Simulation of waste-to-energy (WtE) based on the methane production in Terceira Island. Source: [23].

		Units	Biogas Production 70% CH_4_ (L/unit.day)	Total Biogas 70% CH_4_ (m^3^/day)	Energy (kWh/day)
**Cow Manure**	Cows		550	7700	
**Pig Slurry**	Pigs	1500	100	150	889
**Bird Manure**	Birds	1000	2.5	3	15
**Municipal Solid Waste (MSW)**	People		35	1921	
**Total**	---	---	---	9773	

## Data Availability

The authors declare that data supporting the findings of this study are available within the article.
